# New Face of a Well-Known Hazard: Arsenic Alters H1N1 Response in Mice

**DOI:** 10.1289/ehp.117-a406a

**Published:** 2009-09

**Authors:** Cynthia Washam

**Affiliations:** **Cynthia Washam** writes for *EHP*, *Oncology Times*, and other science and medical publications from South Florida

One of the puzzles of the 2009 pandemic of novel H1N1 influenza virus is why some populations are being hit harder than others—a reminder that known susceptibility factors cannot always explain why otherwise healthy people succumb to diseases that others survive. A team of researchers from Dartmouth Medical School may have uncovered a potential previously unrecognized susceptibility factor, demonstrating that exposure to arsenic significantly weakened mice’s immune response to a mouse-adapted subtype of H1N1 flu **[*EHP*** **117:1441–1447; Kozul et al.]**.

The team believes their study is the first to link flu morbidity to arsenic, which occurs naturally in the drinking water of hundreds of millions of people worldwide. In the United States public drinking water must meet the U.S. Environmental Protection Agency (EPA) arsenic limit of 10 ppb, but private well water is unregulated. Up to 25 million Americans with private wells may be exposed to arsenic levels above the EPA limit. In many regions of the United States and in Mexico, where the novel H1N1 outbreak began, arsenic levels in well water commonly exceed the EPA limit by tenfold or more.

The current study was inspired by recent epidemiologic research indicating that chronic exposure to arsenic increased the risk for a variety of pulmonary diseases including impaired lung function, cancer, and bronchiectasis. Other studies, including recent work by members of this research team [*EHP* 117:1108–1115 (2009)], have indicated that arsenic exposure can suppress the innate immune system. Impairment of the immune cells in the lungs as a result of arsenic exposure could also alter the ability to fight other infectious challenges.

The researchers tested their hypothesis that arsenic could suppress the innate immune response and thereby intensify H1N1 flu infection by giving mice drinking water containing 100 ppb arsenic for 5 weeks. After 5 weeks, the researchers inoculated the arsenic-exposed mice and a group of control mice with the H1N1 virus, and flu morbidity was measured as weight loss.

Control mice experienced moderate weight loss but returned to their original weight by day 16 postinfection. The arsenic-exposed mice had a more dramatic weight loss of up to 20% of their body weight by day 8 postinfection, at which point the researchers euthanized them to prevent suffering, in compliance with institutional animal care standards. In subsequent analyses at day 7 postinfection, examination of the exposed mice’s lungs revealed hemorrhaging, edema, and 10 times more virus than was seen in the lungs of control mice.

Millions of people worldwide are infected with seasonal flu each year, and hundreds of thousands die. Understanding the risk factors that may increase flu cases and deaths could have a potentially significant impact on preventing and treating this common disease.

## Figures and Tables

**Figure:**
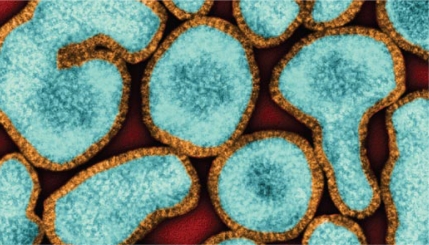
Influenza A virus

